# Comparative analysis of six nutritional scores in predicting prognosis of COVID-19 patients

**DOI:** 10.3389/fnut.2024.1501132

**Published:** 2024-11-28

**Authors:** Shangpu Zou, Pengcheng Lin, Xiaoyu Chen, Lijing Xia, Xiling Liu, Shanshan Su, Ying Zhou, Yuping Li

**Affiliations:** ^1^The Key Laboratory of Interventional Pulmonology of Zhejiang Province, Department of Respiratory and Critical Care Medicine, The First Affiliated Hospital of Wenzhou Medical University, Wenzhou, China; ^2^Department of Respiratory and Critical Care Medicine, The Second Hospital of Yiwu, Yiwu, China

**Keywords:** nutritional scores, COVID-19, prognostic value, nutritional risk, nutritional assessment

## Abstract

**Background:**

Identifying nutritional risk in COVID-19 patients poses a challenge due to the unique qualities of every nutritional screening instrument. The objective was to assess the efficacy of six nutritional scores, including the Nutritional Risk Screening 2002 (NRS-2002) score, the NUTRIC (nutrition risk in the critically ill) score, the modified NUTRIC score, the prognostic nutritional index (PNI), controlling nutritional status (CONUT) score, TCB index (TCBI), predicting prognosis of COVID-19 patients.

**Methods:**

Clinical data were collected from COVID-19 patients admitted to the First Affiliated Hospital of Wenzhou Medical University between December 2022 and February 2023. Participants in this research were divided into two groups: all patients and those specifically from the intensive care unit (ICU). Each group was further stratified into two groups: survivors and non-survivors.

**Result:**

506 COVID-19 patients and 190 COVID-19 patients in intensive care unit (ICU) were evaluated. In all COVID-19 patients, we found that NRS-2002 (*p* < 0.001) and TCBI (*p* = 0.002) were statistically significant independent predictors in multivariate analyses, while APACHE II score (*p* = 0,048) and the mNUTRIC score (*p* = 0.025) were statistically significant independent predictors in multivariate analyses in ICU patients. The NRS-2002 demonstrated a higher AUC value (0.687) than other nutritional scores in all patients, with an optimum cut-off value of 3, translating into a corresponding sensitivity of 66.2% and specificity of 68.7%. With an optimum cut-off value of 4, the mNUTRIC score demonstrated a higher AUC value (0.884) in ICU patients, resulting in a sensitivity of 88.4% and a specificity of 76.9%. By using the discrimination and clinical application (DCA) curve, NRS-2002 demonstrated the greatest net benefit in all patients, while NUTRIC score and mNUTRIC score offered the more significant overall advantage than other nutritional scores in ICU patients. Kaplan–Meier analyses showed lower survival rates in patients in low nutritional risk.

**Conclusion:**

Malnutrition was common in COVID-19 patients. The mNUTRIC score and NRS-2002 were, respectively, more effctive scoring systems of prognosis in all COVID-19 patients and severe or critical COVID-19 patients of the intensive care unit (ICU).

## 1 Introduction

COVID-19 is a critical respiratory illness constituting a substantial threat to human life. There is a great deal of variation in the clinical manifestations of COVID-19, ranging from asymptomatic illness to severe pneumonia with potentially fatal consequences. Acute respiratory distress syndrome (ARDS), multiorgan failure, and sometimes lethal consequences are some of these problems (1–4). Individuals with severe illness had a greater frequency of comorbid diseases than individuals without it. These diseases often need treatment in an intensive care unit (ICU) and may potentially be fatal, thus placing a significant burden on healthcare facilities (5). Early identification of those who might have a serious disease is essential for effective allocation of medical resources and timely intervention to enhance their prognosis. Malnutrition is strongly linked to poor prognosis in various diseases, but is often ignored and modifiable (6–17). By identifying individuals who are at risk of malnutrition, we can offer them early nutritional support, which in turn improves their outcomes and extends their lifespan (9).

In most cases, malnutrition is studied through screening tools, which are usually applied by doctors, nutritionists or other healthcare professionals prior to conducting a comprehensive nutritional assessment. However, there is no golden standard for determining malnutrition (18, 19). There are a few screening tools for malnutrition, which associated with poor prognosis in COVID-19 (20–22). Two scoring systems are used to evaluate the nutritional risk in patients receiving intensive care unit (ICU) treatment: the NUTRIC (nutrition risk in the critically sick) score and the modified NUTRIC score. The sole distinction between the two lies in the examination of interleukin-6 (IL-6) levels (23, 24). The nutritional risk score (NRS-2002) is a commonly utilized nutritional screening tool in clinical settings (25). The prognostic nutritional index (PNI), controlling nutritional status (CONUT) score, and Triglycerides (TG) × Total Cholesterol (TC) × Body Weight (BW) Index (TCBI) are convenient, efficientive and practical nutritional scores that can be derived from readily available and cost-effective parameters (26–28). Nevertheless, determining which scoring system is better at predicting prognostic outcomes for COVID-19 patients still lacks clarity.

There is a dearth of research on how nutritional ratings compare to predict COVID-19 patient prognosis. As a result, we investigated the usefulness of nutrition scores utilizing the various above-mentioned scoring systems in determining the prognostic relevance of the nutritional status in COVID-19 patients.

## 2 Methods

### 2.1 Study population

This single-center, retrospective cohort research included information from 595 patients aged 18 years and above who were under follow-up at the First Affiliated Hospital of Wenzhou Medical University from December 2022 to February 2023.

Patients with positive results from thoracic CT scans or reverse transcriptase-polymerase chain reaction (RT-PCR), which showed features that indicated the disease, were included in the study. The cohort under observation in the ICU comprised individuals diagnosed with severe pneumonia and critically ill patients. The exclusion criteria included re-admissions, immunosuppressed state (treated with corticosteroids, immunosuppressive agents or cytotoxic agents for a duration exceeding 1 month), therapeutic limitations, individuals who are pregnant or whose data is insufficient.

The First Affiliated Hospital Ethics Committee of Wenzhou Medical University approved the research protocol.

### 2.2 Data collection

The baseline information, which includes immunosuppressive and glucocorticoid usage (previous use of immunosuppressive agents and corticosteroids, but for less than 1 month), anthropometric measurements, and demographic traits, history of smoking, chronic medical conditions, days in hospital preceding admission to the ICU, total length of stay in hospital, length of stay in ICU, Glasgow Coma Score (GCS), the requirement for vasopressors, the requirement for noninvasive oxygen treatments and the development of acute kidney damage, the length of invasive mechanical ventilation, the amount of urine produced, the need for renal replacement treatment, disease outcome (survival/non-survival) and laboratory parameters.

### 2.3 Assessment of nutritional status

The NUTRIC score and the mNUTRIC score for each patient were assessed based on six paraments: Acute Physiology and Chronic Health Evaluation II (APACHE II) score, Sequential Organ Failure Assessment (SOFA) score, age, quantity of comorbid conditions, duration of hospital stay prior to ICU admission and interleukin-6 (IL-6) levels (23, 24). NRS-2002 comprises the patient’s declining eating capacity, the recent proportion of weight loss over the last 3 months, the current Body Mass Index (BMI), age and comorbidities (25). PNI was determined using the formula: $PNI=\mathrm{albumin}\;\left(\mathrm{ALB}\right)\;\left(g/L\right)+0.005\times \mathrm{lymphocyte}\;\mathrm{count}\;\left(/mm^{3}\right)$ (26). CONUT was assessed using lymphocyte count, total cholesterol (TC) and $\mathrm{albumin}\;\left(\mathrm{ALB}\right)$ levels (27). TCBI was determined using the formula: $\mathrm{TCBI}=TG\;\left(\mathrm{mg}/\mathrm{dL}\right)\times TC\;\left(\mathrm{mg}/\mathrm{dL}\right)\times BW\;\left(\mathrm{kg}\right)/1000$ (28).

Calculate the NUTRIC score and the mNUTRIC score for patients upon their admission to the ICU, within 24 h of ICU admission, the remaining nutritional scores were calculated within 48 h after the patient is admitted. For the purpose of comparison, instead of using established scoring definitions, we purposely classified patients into low nutritional risk and high nutritional risk based on the optimal cut-off value of the ROC curve for one of any nutritional scores.

### 2.4 Outcome

The rate of COVID-19-related in-hospital mortality was the study’s major endpoint, while the 28-day all-cause mortality was its secondary objective.

### 2.5 Statistical analysis

As a retrospective analysis, there was no prior statistical analysis plan. There was no calculation of statistical power. The missing values for each variable were estimated using multiple imputation (29).

**Figure 1:**
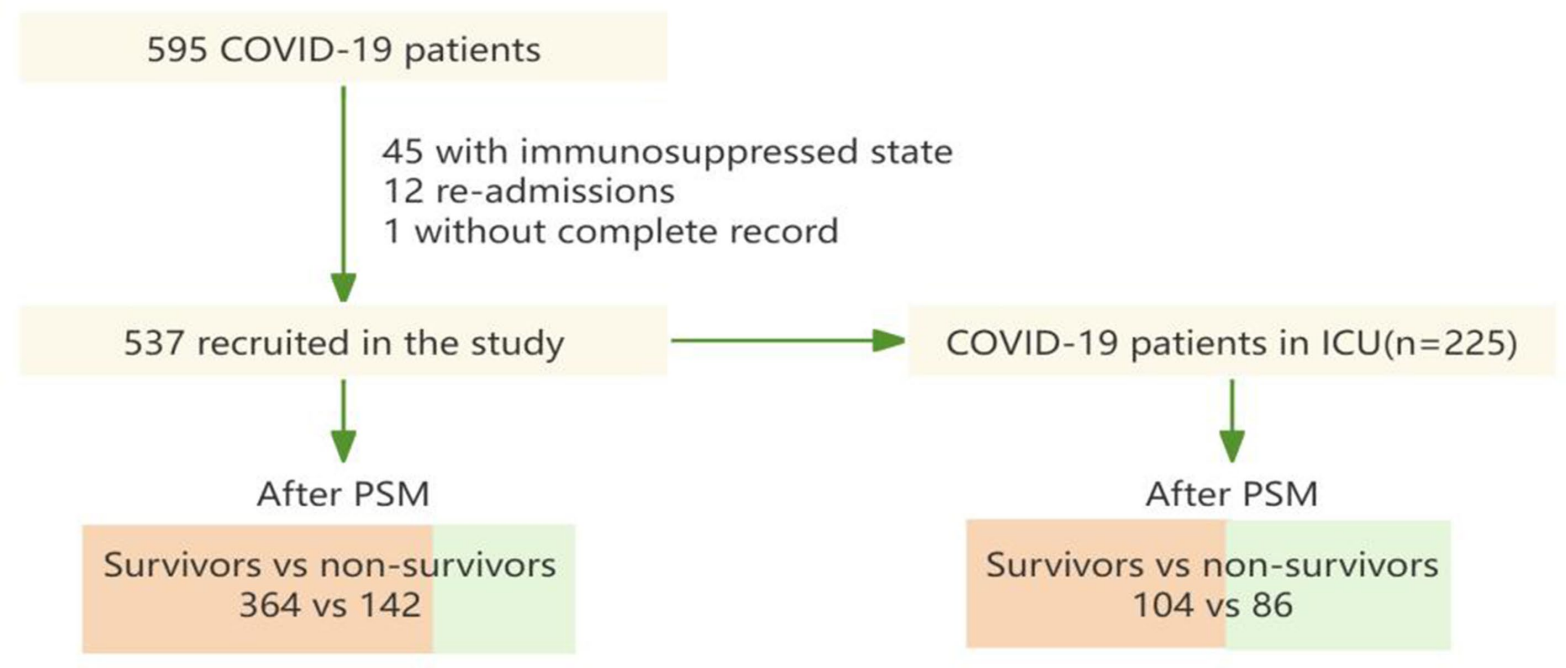
The flowchart showing the strategy of participant enrollment.

In this study, patients were segregated into two distinct groups of study subjects, all patients and ICU patients. Each group was further categorized into survivors and non-survivors (Figure 1). The propensity score-matching (PSM) model is employed to mitigate baseline characteristic disparities among study subjects. For variables that have a normal distribution, the data are shown as mean ± standard deviation; for skewed variables, they are presented as median with interquartile range; and for categorical variables, as *n* (%). In testing, differences were compared using the Mann–Whitney U test for skewed data, the student’s t-test for normally distributed variables, or the Chi-squared test for categorical variables. Spearman’s correlation analysis is used to test the multicollinearity of variables.

The optimum cut-off values of nutritional scores and its area under the curve (AUC) for in-hospital mortality were assessed using receiver operating characteristic (ROC) curves (37). We employed sensitivity, specificity, and accuracy to assess the optimum cut-off values’ diagnostic usefulness. The predictive model proposed here has a net therapeutic benefit that was evaluated using decision-curve analysis (DCA) (38).

Using Kaplan–Meier analyses to ascertain the influence of prognostic variables on patient survival, patients were divided into groups according to their nutritional risk: high and low, which based on the optimal cut-off value derived from the ROC curve of one of any nutritional scores. A significance threshold of *p* < 0.05 was used as the standard for statistical significance in all tests.

R statistical software^1^ was utilized for data handing, statistical analyses, and the generation of graphical representations. The particular packages used were multipleROC, rmda, MatchIt, ggplot2, ggpubr, corrplot.

## 3 Results

### 3.1 Population characteristics

Figure 1 shows that a total of 595 COVID-19 patients were found in the course of the study. Following the exclusion of episodes related to re-admissions (*n* = 12), patients with immunosuppressed state (*n* = 45) and patients without complete record (*n* = 1), 537 patients were included in this study. For subjects enrolled in the propensity score-matching (PSM) model, 142 deceased patients were matched with 364 patients who were discharged. Meanwhile, after PSM, 86 patients in ICU from the dead group were matched to 104 patients in ICU from the discharged group.

Tables 1, 2 respectively described the baseline characteristics of all COVID-19 patients and COVID-19 patients in ICU before and after propensity score matching (PSM). In the respective study groups, the baseline characteristics were found to be comparable between the survivors and the non-survivors after propensity score matching (PSM).

**Table 1:** Baseline characteristics of all COVID-19 patients before and after propensity score matching (PSM).

Variable	Unmatched		*p* value	Matched		*p* value
	Survivors (*n* = 386)	non-survivors (*n* = 151)		Survivors (*n* = 364)	non-survivors (*n* = 142)	
Weight, Mean ± SD	64.49 ± 11.78	63.20 ± 11.66	0.252	64.56 ± 11.74	63.05 ± 11.71	0.195
BMI, Mean ± SD	23.78 ± 3.78	23.07 ± 3.79	0.051	23.75 ± 3.77	23.05 ± 3.80	0.062
Age, M (Q₁, Q₃)	74.00 (65.00, 82.00)	77.00 (71.00, 84.00)	0.002^*^	75.00 (67.00, 83.00)	77.00 (71.00, 84.00)	0.065
Height, M (Q₁, Q₃)	165.00 (158.00, 170.00)	165.00 (160.00, 170.00)	0.275	165.00 (158.75, 170.00)	165.00 (160.00, 170.00)	0.558
Male gender, *n* (%)	255 (66.06)	113 (74.83)	0.049^*^	253 (69.51)	105 (73.94)	0.324
Smoker, *n* (%)	105 (27.20)	43 (28.48)	0.766	103 (28.30)	41 (28.87)	0.897
Glucocorticoid, *n* (%)	38 (9.84)	20 (13.25)	0.254	37 (10.16)	19 (13.38)	0.3
Immunodepressant, *n* (%)	3 (0.78)	0 (0.00)	0.563	3 (0.82)	0 (0.00)	0.563
chronic medical conditions						
Diabetes, *n* (%)	137 (35.49)	58 (38.41)	0.527	133 (36.54)	54 (38.03)	0.755
Hypertension, *n* (%)	217 (56.22)	95 (62.91)	0.157	214 (58.79)	88 (61.97)	0.512
Transplantation, *n* (%)	5 (1.30)	2 (1.32)	1	3 (0.82)	2 (1.41)	0.923
Solid malignancies, *n* (%)	61 (15.80)	19 (12.58)	0.346	58 (15.93)	18 (12.68)	0.357
Hematologic malignancies, *n* (%)	10 (2.59)	8 (5.30)	0.117	8 (2.20)	8 (5.63)	0.089
Cirrhosis, *n* (%)	4 (1.04)	2 (1.32)	1	4 (1.10)	2 (1.41)	1
Hepatitis, *n* (%)	7 (1.81)	1 (0.66)	0.553	5 (1.37)	1 (0.70)	0.867
Heart Failure, *n* (%)	3 (0.78)	4 (2.65)	0.195	3 (0.82)	3 (2.11)	0.456
Nephropathy, *n* (%)	28 (7.25)	22 (14.57)	0.009^*^	27 (7.42)	13 (9.15)	0.515
Stroke, *n* (%)	33 (8.55)	22 (14.57)	0.039^*^	33 (9.07)	18 (12.68)	0.226
COPD, *n* (%)	10 (2.59)	5 (3.31)	0.869	10 (2.75)	4 (2.82)	1
Bronchiectasis, *n* (%)	1 (0.26)	0 (0.00)	1	1 (0.27)	0 (0.00)	1
Asthma, *n* (%)	1 (0.26)	1 (0.66)	0.484	1 (0.27)	1 (0.70)	0.483
The history of Pneumonia, *n* (%)	15 (3.89)	2 (1.32)	0.211	15 (4.12)	2 (1.41)	0.212
Interstitial lung disease, *n* (%)	2 (0.52)	2 (1.32)	0.675	2 (0.55)	2 (1.41)	0.673

BMI, body mass index; COPD, chronic obstructive pulmonary disease.

Data are shown as *n* (%), mean ± standard deviation (SD) or median with interquartile range. In testing, differences were compared using the Mann–Whitney U test for skewed data, the student’s *t*-test for normally distributed variables, or the Chi-squared test for categorical variables. *p* value < 0.05 is statistically significant. *Indicates *p* value < 0.05.

**Table 2:** Baseline characteristics of COVID-19 patients in ICU before and after propensity score matching (PSM).

Variable	Unmatched		*p* value	Matched		*p* value
	Survivors (*n* = 112)	Non-survivors (*n* = 113)		Survivors (*n* = 104)	Non-survivors (*n* = 86)	
Weight, Mean ± SD	65.21 ± 11.00	63.55 ± 12.17	0.283	64.60 ± 10.66	63.49 ± 12.29	0.505
BMI, Mean ± SD	23.71 ± 3.58	23.20 ± 3.95	0.308	23.61 ± 3.58	23.26 ± 3.94	0.522
Age, M (Q₁, Q₃)	75.00 (66.00,81.00)	77.00 (71.00, 84.00)	0.031^*^	76.00 (68.00, 82.25)	76.50 (71.00, 82.00)	0.481
Height, M (Q₁, Q₃)	167.00 (160.00, 170.00)	166.00 (160.00, 170.00)	0.738	167.00 (160.00, 170.00)	165.00 (160.00, 170.00)	0.779
Male gender, *n* (%)	73 (65.18)	84 (74.34)	0.135	68 (65.38)	60 (69.77)	0.521
Smoker, *n* (%)	26 (23.21)	32 (28.32)	0.381	25 (24.04)	21 (24.42)	0.951
Glucocorticoid, *n* (%)	12 (10.71)	14 (12.39)	0.694	11 (10.58)	12 (13.95)	0.478
Immunodepressant, *n* (%)	1 (0.89)	0 (0.00)	0.498	1 (0.96)	0 (0.00)	1
chronic medical conditions						
Diabetes, *n* (%)	42 (37.50)	45 (39.82)	0.721	40 (38.46)	38 (44.19)	0.425
Hypertension, *n* (%)	63 (56.25)	67 (59.29)	0.644	61 (58.65)	49 (56.98)	0.816
Transplantation, *n* (%)	1 (0.89)	2 (1.77)	1	1 (0.96)	1 (1.16)	1
Solid malignancies, *n* (%)	19 (16.96)	13 (11.50)	0.241	19 (18.27)	10 (11.63)	0.205
Hematologic malignancies, *n* (%)	0 (0.00)	7 (6.19)	0.022^*^	0 (0.00)	0 (0.00)	–
Cirrhosis, *n* (%)	1 (0.89)	2 (1.77)	1	1 (0.96)	2 (2.33)	0.868
Hepatitis, *n* (%)	3 (2.68)	1 (0.88)	0.608	2 (1.92)	1 (1.16)	1
Heart Failure, *n* (%)	1 (0.89)	2 (1.77)	1	1 (0.96)	2 (2.33)	0.868
Nephropathy, n (%)	11 (9.82)	13 (11.50)	0.683	10 (9.62)	12 (13.95)	0.352
Stroke, *n* (%)	16 (14.29)	17 (15.04)	0.872	16 (15.38)	12 (13.95)	0.782
COPD, *n* (%)	6 (5.36)	4 (3.54)	0.735	6 (5.77)	4 (4.65)	0.986
Bronchiectasis, *n* (%)	1 (0.89)	0 (0.00)	0.498	1 (0.96)	0 (0.00)	1
Asthma, *n* (%)	2 (1.79)	0 (0.00)	0.247	1 (0.96)	0 (0.00)	1
The history of Pneumonia, *n* (%)	5 (4.46)	0 (0.00)	0.069	5 (4.81)	0 (0.00)	0.108
Interstitial lung disease, *n* (%)	2 (1.79)	2 (1.77)	1	2 (1.92)	2 (2.33)	1

BMI, body mass index; COPD, chronic obstructive pulmonary disease; ICU, intensive care unit.

Data are shown as *n* (%), mean ± standard deviation (SD) or median with interquartile range. In testing, differences were compared using the Mann–Whitney U test for skewed data, the student’s *t*-test for normally distributed variables, or the Chi-squared test for categorical variables. *p* value < 0.05 is statistically significant. *Indicates *p* value < 0.05.

Among the 537 selected patients, their median age ranged from 68.00 to 83.00 years, with 368 male and 169 female cases. Among them, 151 patients died, 386 patients were discharged, and 225 patients were admitted in ICU. The mortality rate inside hospitals was reported to be 28.1%, the ICU admission rate was 37.8%. The participants who died were older (77.0 versus 74.0 years of age, *p* = 0.002), had a greater percentage of men and had higher prevalence of chronic medical conditions, including chronic kidney diseases (14.57% versus 7.25%, *p* = 0.009), the history of stroke (14.57% versus 8.55%, *p* = 0.039) than those who were discharged.

The baseline characteristics of all COVID-19 patients and COVID-19 patients in ICU before and after propensity score matching were shown in Tables 1, 2.

### 3.2 Testing multicollinearity of variables

**Figure 2:**
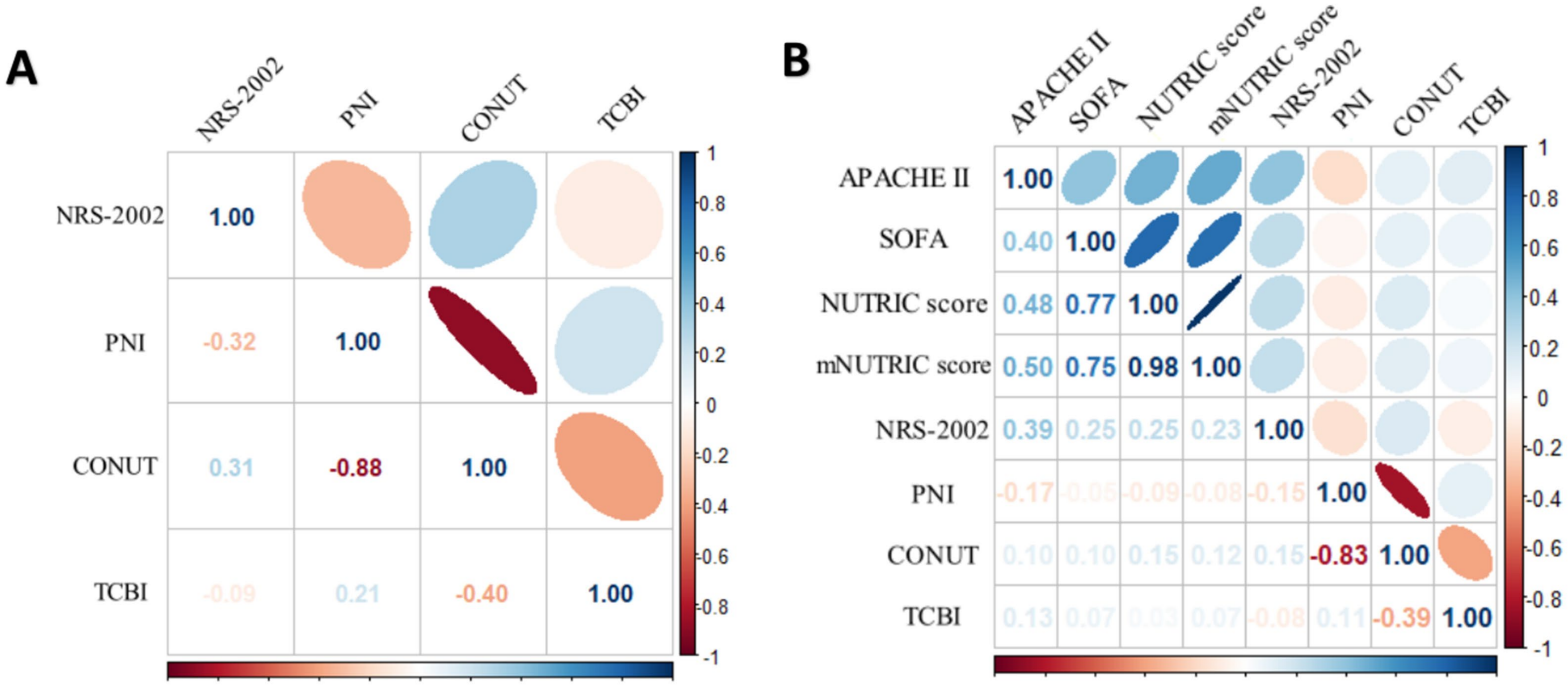
**(A)** Spearman correlation analysis of NRS-2002, PNI CONUT and TCBI for all COVID-19 patients. **(B)** Spearman correlation analysis of the NUTRIC score, mNUTRIC score and its components, NRS-2002, PNI, CONUT, TCBI for all COVID-19 patients.

PNI and CONUT showed a strong negative association, according to Spearman’s correlation study (*r* = −0.88) in all COVID-19 patients. In COVID-19 patients in ICU, apart from the mNUTRIC score and the NUTRIC score exhibited a strong correlation, the mNUTRIC score and its components showed a strong positive correlation, PNI and CONUT showed a strong negative association, according to spearman’s correlation study (*r* = −0.83) (Figure 2).

### 3.3 Comparison of predictive accuracy and predictors for in-hospital mortality

Univariable and multivariable analyses of in-hospital death in all patients and ICU patients are detailed in Tables 3, 4, respectively.

**Table 3:** Univariable and multivariable analysis of in-hospital death in all COVID-19 patients.

	Univariable analysis					Multivariable analysis				
Variables	Beta	SE	*Z*	OR (95%CI)	*p* value	aBeta	aSE	aZ	aOR (95%CI)	a*p* value
NRS-2002	0.44	0.07	6.34	1.55 (1.35–1.77)	<0.001^*^	0.39	0.07	5.19	1.47 (1.27–1.70)	<0.001^*^
PNI	−0.07	0.02	−4.24	0.93 (0.90–0.96)	<0.001^*^	0.01	0.04	0.26	1.01 (0.94–1.08)	0.796
CONUT	0.17	0.04	4.13	1.18 (1.09–1.28)	<0.001^*^	0.18	0.09	1.95	1.19 (1.00–1.42)	0.051
TCBI	0	0	2.09	1.01 (1.01–1.01)	0.037^*^	0	0	3.14	1.01 (1.01–1.01)	0.002^*^
LOS	0.02	0.01	1.99	1.02 (1.01–1.04)	0.047^*^	0	0.01	−0.07	1.00 (0.98–1.02)	0.944

SE, standard error; OR, odds ration; CI, confidence interval; NRS-2002, the Nutritional Risk Screening 2002; PNI, the prognostic nutritional index; CONUT, controlling nutritional status; TCBI, Triglycerides × Total Cholesterol × Body Weight Index; LOS, length of hospital.

In testing, differences were compared using the Binary Logistic Regression Analysis. *p* value < 0.05 is statistically significant. *Indicates *p* value < 0.05.

**Table 4:** Univariable and multivariable analysis of in-hospital death in COVID-19 patients in ICU.

	Univariable analysis					Multivariable analysis				
Variables	Beta	SE	*Z*	OR (95%CI)	*p* value	aBeta	aSE	aZ	aOR (95%CI)	a*p* value
APACHE II	0.14	0.04	3.69	1.15(1.07–1.25)	<0.001	−0.13	0.07	−1.98	0.88 (0.77–0.99)	0.048
SOFA	0.36	0.06	5.92	1.43(1.27–1.61)	<0.001	−0.06	0.09	−0.69	0.94 (0.80–1.12)	0.489
NUTRIC score	1.33	0.19	7.08	3.78 (2.61–5.46)	<0.001	0.27	0.6	0.44	1.31 (0.40–4.23)	0.656
mNUTRIC score	1.47	0.21	6.99	4.36(2.89–6.59)	<0.001	1.47	0.66	2.24	4.36 (1.20–15.77)	0.025
NRS-2002	0.26	0.1	2.71	1.30 (1.08–1.58)	0.007	0.19	0.15	1.27	1.20 (0.90–1.60)	0.203
PNI	−0.01	0.03	−0.28	0.99 (0.94–1.04)	0.78					
CONUT	0.03	0.06	0.51	1.03(0.91–1.17)	0.609					
TCBI	0	0	1.8	1.00(1.00–1.00)	0.073					
LOS	−0.01	0.01	−1.2	0.99 (0.97–1.01)	0.231					
LOS in ICU	0	0.01	−0.36	1.00 (0.97–1.02)	0.722					

SE, standard error; OR, odds ration; CI, confidence interval; APACHE II, acute physiology and chronic health evaluation II; SOFA, sequential organ Failure assessment; NUTRIC score, the nutrition risk in the critically ill score; mNUTRIC score, the modified nutrition risk in the critically ill score; NRS-2002, the Nutritional Risk Screening 2002; PNI, the prognostic nutritional index; CONUT, controlling nutritional status; TCBI, Triglycerides × Total Cholesterol × Body Weight Index; LOS, length of hospital; ICU, intensive care unit.

In testing, differences were compared using the Binary Logistic Regression Analysis. *p* value < 0.05 is statistically significant. *Indicates *p* value < 0.05.

Table 3 indicated, that in all COVID-19 patients, NRS-2002(*p* < 0.001), CONUT (p < 0.001), PNI (*p* < 0.001), LOS (*p* = 0.047) were significantly elevated in non-survivors compared to survivors, PNI (p < 0.001) was much greater in survivors than in non-survivors. However, TCBI although was an independent predictor in multivariate analyses that was statistically significant, but its Beta was 0. We also found that NRS-2002 (*p* < 0.001) and TCBI (*p* = 0.002) were statistically significant independent predictors in multivariate analyses.

Table 4 indicated that among patients in ICU, APACHE II score (*p* < 0.001), SOFA score (*p* < 0.001), the NUTRIC score (*p* < 0.001), the mNUTRIC score (*p* < 0.001), NRS-2002 (*p* = 0.007) were much greater in non-survivors than in survivors. The mNUTRIC score (*p* = 0.025) and the APACHE II score (*p* = 0.048) were both shown to be statistically significant independent predictors in multivariate analyses.

**Figure 3:**
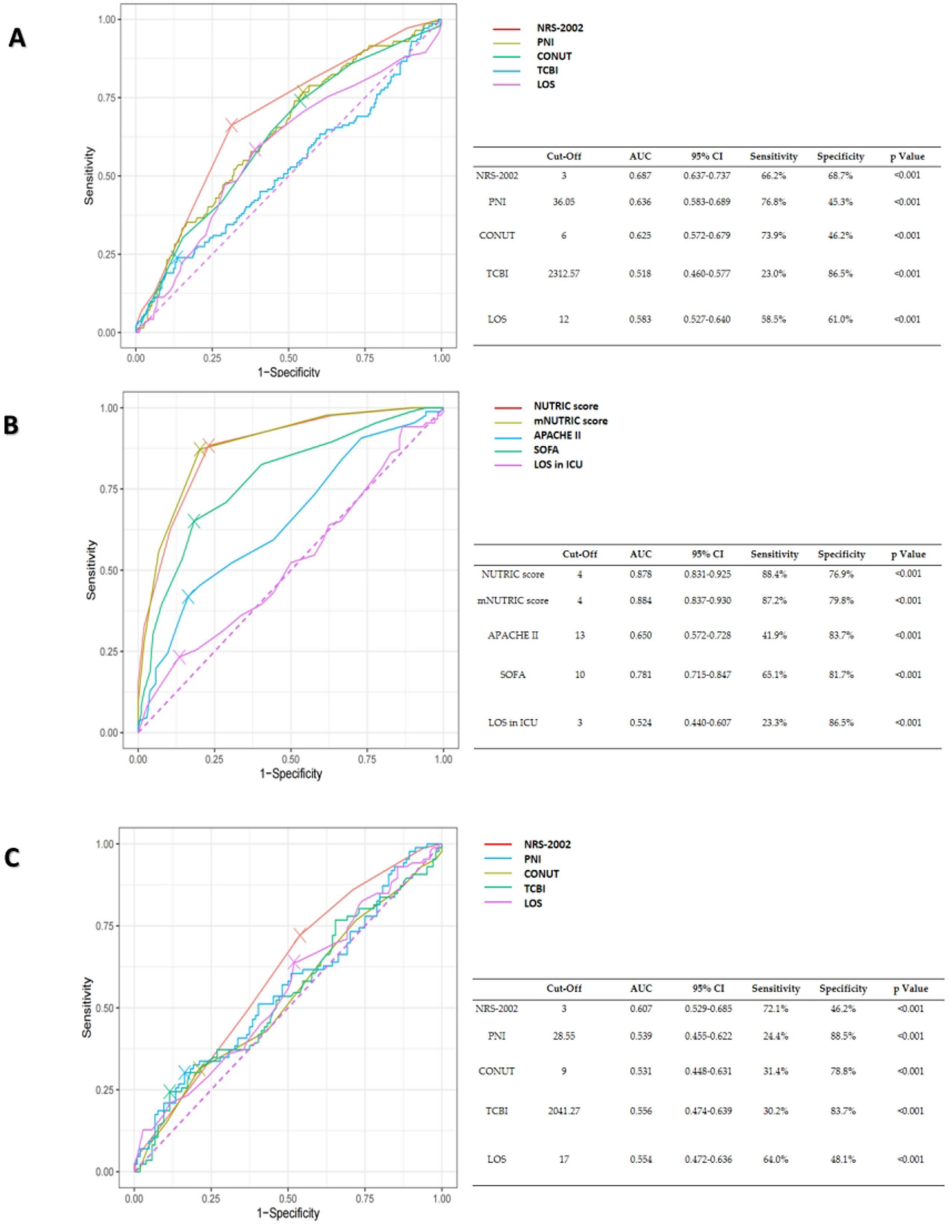
**(A)** The ROC curves of NRS-2002, PNI, CONUT, TCBI and hospital length of stay (LOS) for the prediction of in-hospital mortality of all COVID-19 patients. **(B)** The ROC curves of the NUTRIC score, the mNUTRIC score and its components, hospital length of stay (LOS) in ICU for the prediction of in-hospital mortality of COVID-19 patients in ICU. **(C)** The ROC curves of NRS-2002, PNI, CONUT, TCBI, hospital length of stay (LOS) for the prediction of in-hospital mortality of COVID-19 patients in ICU.

Employing the ROC approach, curves were generated to ascertain the optimal cut-off points for forecasting mortality inside hospitals. The optimum cut-off value for the CONUT score was 6, which produced an AUC of 0.625, 73.9% sensitivity, and 46.2% specificity for all patients. With the optimum cut-off value set at 3, the NRS-2002 score demonstrated a higher AUC value of 0.687, surpassing the CONUT score and yielding a sensitivity and specificity of 66.2 and 68.7%, respectively. The sensitivity and specificity of the APACHE II score in ICU patients were 41.9 and 83.7%, respectively, with an optimum cut-off value of 13 and an AUC of 0.65. With an optimum cut-off value of 4, the mNUTRIC score and the NUTRIC score showed surperior AUC values of 0.884 and 0.878, respectively, outperforming the APACHE II score (Figure 3).

Statistical significance calculated by De-long’s test, the outcomes are shown in Table 5.

**Table 5:** De-long’s test of AUC of nutritional scores in all COVID-19 patients and COVID-19 patients in ICU.

All COVID-19 patients				COVID-19 patients in ICU			
Variables	AUC	95% CI	*p*-value	Variables	AUC	95% CI	*p*-value
NRS-2002	0.687	0.637–0.737	Ref.	mNUTRIC score	0.884	0.837–0.930	Ref.
PNI	0.636	0.583–0.689	0.125	NUTRIC score	0.878	0.831–0.925	0.456
CONUT	0.625	0.572–0.679	0.066	APACHE II	0.650	0.572–0.728	<0.001^*^
TCBI	0.518	0.460–0.577	<0.001^*^	SOFA	0.781	0.715–0.847	<0.001^*^
LOS	0.583	0.527–0.640	0.002^*^	NRS-2002	0.607	0.529–0.685	<0.001^*^
				PNI	0.539	0.455–0.622	<0.001^*^
				CONUT	0.531	0.448–0.631	<0.001^*^
				TCBI	0.556	0.474–0.639	<0.001^*^
				LOS	0.554	0.472–0.636	<0.001^*^
				LOS in ICU	0.524	0.440–0.607	<0.001^*^

AUC, area under curve; CI, confidence interval; ICU, intensive care unit; NRS-2002, the Nutritional Risk Screening 2002; PNI, the prognostic nutritional index; CONUT, controlling nutritional status; TCBI, Triglycerides × Total Cholesterol × Body Weight Index; LOS, length of hospital; mNUTRIC score, the modified nutrition risk in the critically ill score; NUTRIC score, the nutrition risk in the critically ill score; APACHE II, acute physiology and chronic health evaluation II; SOFA, sequential organ Failure assessment.

In testing, differences were compared using the De-long’s test. *p* value < 0.05 is statistically significant. *Indicates *p* value < 0.05.

### 3.4 Comparison of clinical application value

**Figure 4:**
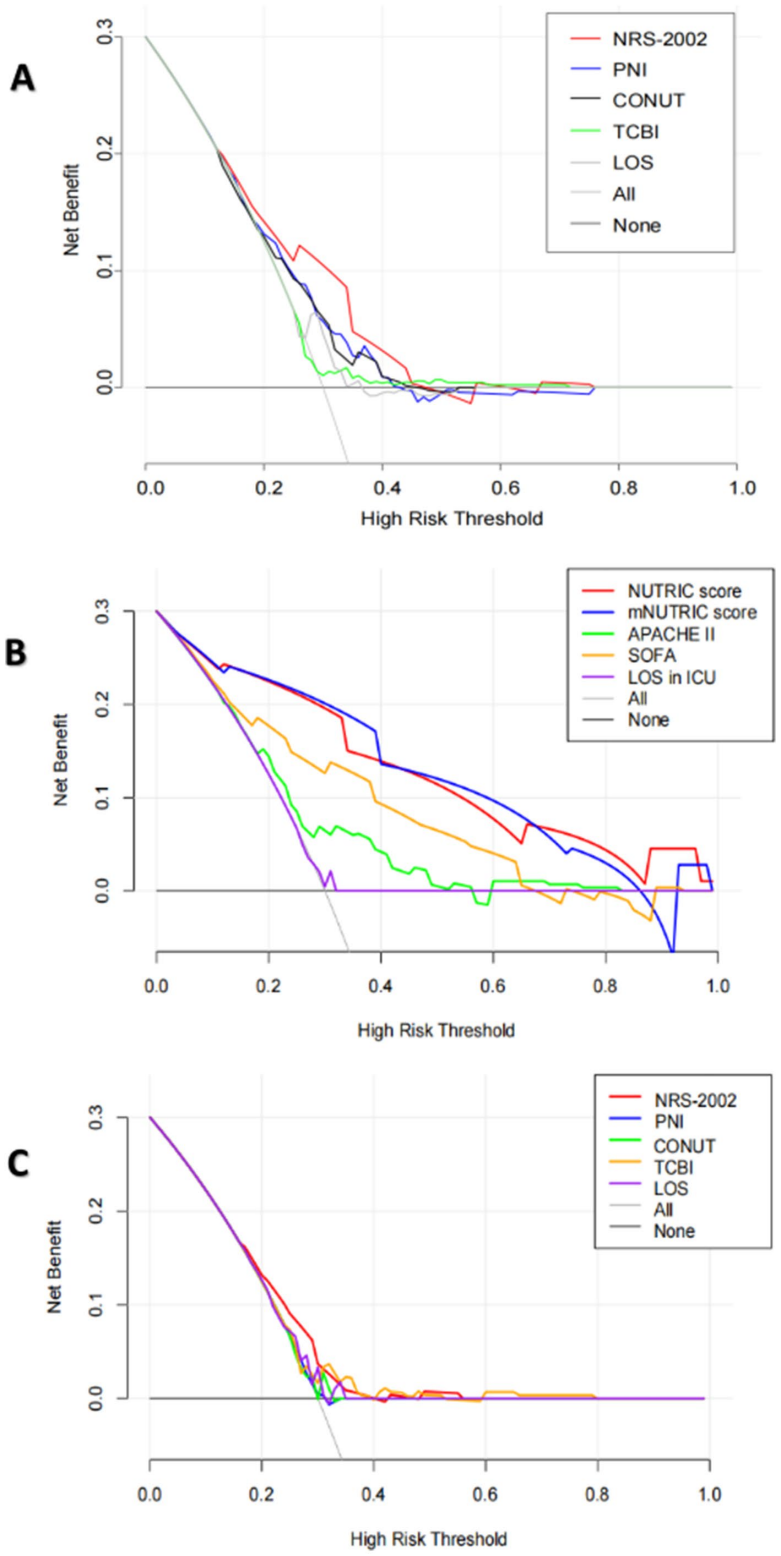
**(A)** The DCA curves of NRS-2002, PNI, CONUT, TCBI and hospital length of stay (LOS) for the prediction of in-hospital mortality of all COVID-19 patients. **(B)** The DCA curves of the NUTRIC score, the mNUTRIC score and its components, LOS in ICU for the prediction of in-hospital mortality of COVID-19 patients in ICU. **(C)** The DCA curves of NRS-2002, PNI, CONUT, TCBI, hospital length of stay (LOS), LOS in ICU for the prediction of in-hospital mortality of COVID-19 patients in ICU.

The Discrimination and Clinical Application (DCA) curve was shown in Figure 4. In all patients, through a comparison of NRS-2002 with PNI, CONUT score, TCBI, and length of hospital (LOS), it was evident that NRS-2002 demonstrated the greatest net benefit. In ICU patients, upon evaluating the NUTRIC score, the mNUTRIC score, APACHE 2 score, SOFA score, NRS-2002, PNI, TCBI, and CONUT scores, LOS, LOS in ICU, it becomed apparent that NUTRIC score and mNUTRIC score offered the more significant overall advantage than other nutritional scores.

### 3.5 Kaplan–Meier analyses showed lower survival rates in patients in low nutritional risk

**Figure 5:**
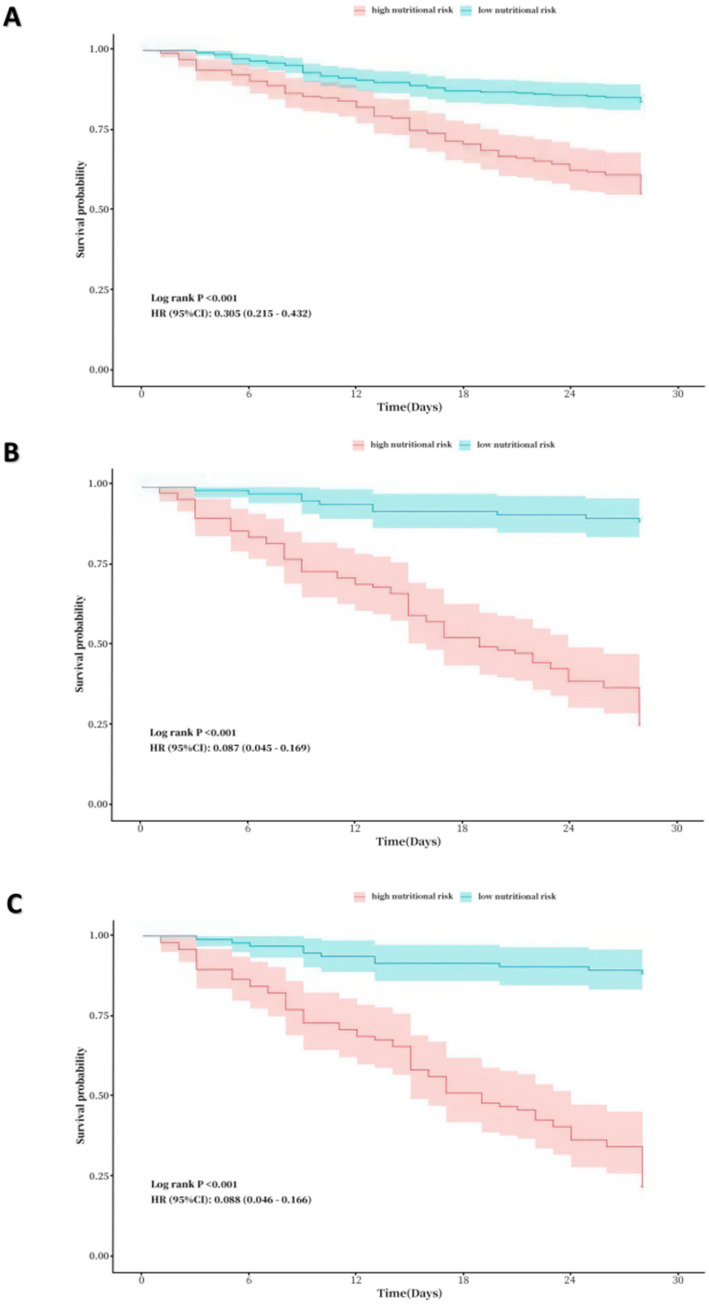
**(A)** Kaplane-Meier curve for 28-day all-cause mortality by nutritional risk in all patients. (assessed by NRS-2002, where low nutrional risk: NRS-2002 < 3 vs. high nutritional risk: NRS-200 ≥ 3). **(B)** Kaplane-Meier curve for 28-day all-cause mortality by nutritional risk in ICU patients (assessed by the NUTRIC score, where low nutrional risk: the NUTRIC score < 4 vs. high nutritional risk: the NUTRIC score ≥ 4). **(C)** Kaplane-Meier curve for 28-day all-cause mortality by nutritional risk in ICU patients (assessed by the mNUTRIC score, where low nutrional risk: the NUTRIC score < 4 vs. high nutritional risk: the NUTRIC score ≥ 4).

In all COVID-19 patients, 208 individuals had high nutritional risk (41.1%), which assessed by NRS-2002. In COVID-19 patients in ICU, 94 individuals had high nutritional risk (49.5%). Figure 5 shows the Kaplane Meier curve for 28-day all-cause mortality stratified by nutritional risk, as evaluated using NRS-2002 in all COVID-19 patients, as well as the NUTRIC score and the mNUTRIC score among COVID-19 patients in ICU. Patients with a high risk of malnutrition exhibit a significantly poorer prognosis than those at low nutritional risk, with the statistical significance of the observed discrepancies (*p* < 0.001).

## 4 Discussion

The COVID-19 pandemic has brought about various challenges and posed threats to both healthcare and economic infrastructures (5, 30). A few studies have demonstrated the importance of nutritional scores to forecast the prognosis of COVID-19 patients, highlighting its ability to promptly detect malnutrition (20–22). Currently, the incidence of malnutrition may vary depending on the nutritional screening methods used, and there is no widely agreed set of criteria for identifying malnutrition (18, 19). To the extent of our understanding, this study represents the initial investigation into the prognostic significance of six nutritional scores among patients diagnosed with COVID-19. The current investigation suggested that patients identified as having a high nutritional risk based on any of the six objective nutritional scores (the NUTRIC score, the mNUTRIC score, NRS-2002, PNI, TCBI, and CONUT score), demonstrated lower overall survival rates. Furthermore, NRS-2002 and TCBI were connected with significant in-hospital mortality in each patient in an independent manner, APACHE 2 score and mNUTRIC score were independently correlated with high in-hospital mortality in ICU patients, even after controlling for irrelevant factors through binary logistic regression analysis, the conclusion remains valid. As an independent predictor in multivariate analyses, TCBI was statistically significant in all COVID-19 patients, but its Beta was 0. The reason maybe was the insufficient sample size.

In ICU patients, the AUC values of both the mNUTRIC score and the NUTRIC score were significantly higher than those of other nutritional scores, indicating superior predictive accuracy. In all COVID-19 patients, the NRS-2002 score exhibited a higher AUC value compared to other nutritional scores, despite not showing significant differences from them. Evaluating the clinical application value of various nutritional scores through DCA Curves, NRS-2002 showed the greatest net benefit in all patients, the NUTRIC score and the mNUTRIC score were offer significant advantage over other nutritional indices in ICU patients. The results above indicated that the mNUTRIC score and the NUTRIC score exhibited more effective prognostic assessment capabilities among all COVID-19 patients, while NRS-2002 score exhibited more effective prognostic assessment capability in COVID-19 patients of ICU. Further Kaplan–Meier analyses for these three factors reaffirmed their predictive significance for patient prognosis.

Several potential factors may contribute to the risk of malnutrition among COVID-19 patients. The development of malnutrition is mainly influenced by multiple factors, including diminished dietary intake, elevated requirements for energy and protein, augmented losses, and inflammation (31). Elsa Dent noted that aging, inadequate supply of food, socioeconomic and psychological factors, and modifiable risk factors such as low physical function, low appetite, eating dependency, poor self-perceived health, a previous hospital stay, marital status, and poor oral health. These elements collectively contribute significantly to the occurrence of malnutrition (32).

In 2016, the American Society for Parenteral and Enteral Nutrition (ASPEN) endorsed the utilization of both NRS-2002 and the NUTRIC score for evaluating the nutritional status of critically ill patients (33). The NUTRIC score considers inflammation and nutritional factors, including age, BMI, organ dysfunction, diagnosis, inflammatory markers and nutritional therapy requirements. On the other hand, NRS-2002 takes into account the patient’s nutritional status, age, BMI, nature and severity of the disease and treatment modalities. Both of them share the APACHE II score as a common variable, which can be used to forecast the severity of diseases and assist researchers in evaluating the effectiveness of new or alternative treatments (33). Neeraj Kumar observed a mortality rate of 92.8% in COVID-19 patients with elevated NUTRIC score, contrasting sharply with a 38% mortality rate in patients with low NUTRIC score, while using 3.5 as the cut-off value (34).

Berkay Kucuk reported that, in addition to the APACHE II and SOFA scores, the NUTRIC score and the mNUTRIC score were both effective in predicting death in COVID-19 patients in the intensive care unit, which is consistent with our research (22). Considering its easier accessibility, the mNUTRIC score may be preferred in comparison to the NUTRIC score. The research conducted by Matteo Luigi Giuseppe Leoni indicates that the prevalence of malnutrition is high among critically ill COVID-19 patients admitted to the ICU, and that mNUTRIC and CRP levels are independently associated with 28-day mortality in critically ill COVID-19 patients (35). In our presented study, we determined that the mNUTRIC score cut-off value of 4 for predicting in-hospital mortality in COVID-19 patients was much lower than the previously established cut-off of 5. This suggested an increased importance of nutrition in COVID-19 patients relative to those unaffected by the SARS-CoV-2, necessitating a paramount focus on this aspect.

Ghalia Shamlan observed that COVID-19 patients with an infection duration of 6 months or longer, those who had been vaccinated, obese patients, and those without cardiovascular disease were less likely to experience malnutrition when assessed using the NRS-2002 as a tool for evaluating nutritional risk (36). Ghadamieh Fatemeh et al. reported that patients exhibiting elevated NRS-2002 scores experienced increased in-hospital mortality rates, prolonged hospital stays, and higher rates of ICU admission (20). Among all COVID-19 patients, we found that the NRS-2002 demonstrated superior predictive efficacy for in-hospital mortality compared to other nutritional assessment scores, which also served as a stand-alone predictor of death inside a hospital. The NRS-2002 was not specifically intended for the assessment of critically ill patients. In COVID-19 patients of ICU, the NUTRIC and mNUTRIC scores have much more prediction power for in-hospital mortality than the NRS-2002 score. Interestingly, Audrey Machado dos Reis also demonstrated that the mNUTRIC exhibited superior discriminatory performance in calculating the critical illness patients’ chance of dying in the hospital (8).

Our investigation is constrained by specific limitations. First, all score data were gathered exclusively by a single trained investigator. Second, this research is a retrospective study, hence susceptible to inherent limitations common in such studies. Objective data like laboratory values may be underrepresented due to reliance on historical medical records predating the study. Third, this study, conducted at a single center, presents both benefits and limitations inherent to its design. Enrolling numerous consecutive patients and ensuring uniform criteria and assessments are simpler in a single center. Conversely, variations in patients and procedures, along with potential enrollment challenges, might compromise the epidemiological representativeness of multicenter studies compared to a well-executed single-center study. Our study has several innovative aspects. First, following PSM based on baseline characteristics, we reduced selection bias and enhancing the credibility of causal inference. Furthermore, we divided the study population into all patients and ICU patients, comparing more comprehensively the predictive abilities of prognosis of various nutritional scores. On the other hand, it is underscored that this study represents the inaugural evaluation of the performance of six nutritional scores to date, to predict prognosis of COVID-19 patients that could be implemented to improve prognosis in this population.

## 5 Conclusion

This study revealed that malnutrition is prevalent among COVID-19 patients. The mNUTRIC score and NRS-2002 were, respectively, more effctive scoring systems of prognosis in all COVID-19 patients and COVID-19 patients of ICU. Early application of the aforementioned nutritional scores in clinical practice to evaluate nutritional risk in COVID-19 patients, it may be possible to identify the risk of malnutrition earlier and implement nutritional interventions, thereby reducing mortality rates and alleviating the socioeconomic burden. It is anticipated that this study will be expanded to include a larger population.

## Data Availability

The original contributions presented in the study are included in the article/supplementary material, further inquiries can be directed to the corresponding authors.

## Footnotes

^1^ https://www.Rproject.org/
